# miR-654-5p Contributes to the Activation and Proliferation of Hepatic Stellate Cells by Targeting RXRα

**DOI:** 10.3389/fcell.2022.841248

**Published:** 2022-04-06

**Authors:** Heming Ma, Xiaomei Wang, Xu Liu, Chang Wang, Xiuzhu Gao, Junqi Niu

**Affiliations:** ^1^ Department of Hepatology, The First Hospital of Jilin University, Changchun, China; ^2^ Key Laboratory of Organ Regeneration and Transplantation of Ministry of Education, The First Hospital of Jilin University, Changchun, China

**Keywords:** RXRα, miR-654-5p, hepatic stellate cells, liver fibrosis, target

## Abstract

Liver fibrosis (LF) is a major disease that threatens human health. Hepatic stellate cells (HSCs) contribute directly to LF via extracellular matrix (ECM) secretion. Moreover, RXRα is an important nuclear receptor that plays a key regulatory role in HSC activation. Meanwhile, microRNAs (miRNAs) have been identified as significant regulators of LF development. In particular, miR-654-5p is involved in cellular migration and proliferation, and *via* bioinformatics analysis, has been identified as a potential factor that targets RXRα in humans and in mice. However, the precise relationship between miR-654-5p and RXRα in the context of LF, remains unknown and is the primary focus of the current study. To establish *in vitro* activated cell model human primary HSCs were cultured *in vitro* and LX-2 cells were stimulated with recombinant human TGF-β1. mRNA and protein levels of RXRα, miR-654-5p and fibrogenic genes were compared in quiescent and activated HSCs. Moreover, after transfected with miR-654-5p mimics, the expression changes of above related genes in LX-2 cells were estimated. Meanwhile, cell proliferation and apoptosis were detected in miR-654-5p overexpressed LX-2 cells. Simultaneously, the targeted binding between miR-654-5p and RXRα was verified in LX-2 cells. Carbon tetrachloride (CCl_4_)-induced mouse model with liver fibrosis was use to research the role of the miR-654-5p *in vitro*. Our results show that miR-654-5p expression levels increased in activated human HSCs and TGFβ-treated LX-2 cells. Moreover, miR-654-5p mimics markedly promoted LX-2 cell proliferation while inhibiting their apoptosis. Accordingly, the expression levels of RXRα are decreased in activated HSCs and LX-2 cells. Additionally, dual-luciferase reporter assay results reveal direct targeting of RXRα by miR-654-5p. Similarly, *in vivo* miR-654-5p overexpression aggravates LF in mice that are intraperitoneally injected with CCl_4_. Taken together, our findings elucidated a novel molecular mechanism with potential use for treatment of LF.

## Introduction

Liver fibrosis (LF) is a dynamic and reversible pathological process. Uncontrolled LF progresses to cirrhosis and even hepatocellular carcinoma (HCC), a major disease threatening human health (Tao et al., 2020). LF is characterized by extracellular matrix (ECM) accumulation, a process that is closely associated with hepatic stellate cells (HSCs). Transdifferentiation of quiescent HSCs into a myofibroblast-like cells is referred to as “activation” (Tsuchida and Friedman, 2017). In fibrotic livers, activated HSCs can proliferate, migrate, and contract, while also secreting a large amount of ECM, tissue inhibitors of metalloproteinases (TIMPs), and matrix metalloproteinases (MMPs), all of which play key roles in LF (Ezhilarasan et al., 2018; Roeb, 2018). Hence, maintenance of HSC quiescence may serve to resolve LF.

MicroRNAs (miRNAs) are ∼22 nt long, single-stranded small non-coding RNAs that regulate various cellular biological processes, including cell proliferation, apoptosis, and differentiation (Tsai and Yu, 2010). As such have summarized several miRNAs involved in the regulation of LF through HSCs (Ezhilarasan, 2020; Fang et al., 2021). In particular, microRNA-654-5p (miR-654-5p) is involved in autophagy and inflammatory signaling pathways (Kong, 2020; Li et al., 2021a; Wang et al., 2021) and regulates the proliferation and migration of various tumor cells (Tan et al., 2016; Lu et al., 2018; Huang et al., 2020; Xu et al., 2020; Zhang et al., 2020). Meanwhile, inhibition of the miR-654-5p/SMAD2 axis induces HCC cell proliferation, invasion, and migratio (Lu et al., 2021). However, the role of miR-654-5p in HSC activation within the context of LF, as well as the underlying potential molecular mechanism remain to be elucidated.

Retinoic acid (RA), the main active metabolite of vitamin A, has a key role in several essential biological processes, including embryogenesis, organogenesis, cell proliferation, differentiation, and apoptosis. The biological effects of RA are primarily mediated by retinoid receptors (RRs), including retinoid acid receptors (RARs) and retinoid X receptors (RXRs). RRs regulate gene transcription after binding to retinoic acid response elements (RAREs) in the target gene promoter region (Abdel-Bakky et al., 2020; Li et al., 2021b). RRs are expressed in quiescent HSCs in rodents and humans. RXRs have three subtypes: RXR-α, -β, and -γ, of which RXRα is the most expressed subtype on HSCs. However, the expression of RXRα decreases following HSC activation (Vogel et al., 2000). Specifically, bioinformatics analysis demonstrated that RXRα expression is downregulated in the livers of patients with liver cirrhosis caused by hepatitis B virus (HBV), hepatitis C virus, and nonalcoholic fatty liver disease (NAFLD), and is also downregulated in HSCs that are activated *in vivo* in carbon tetrachloride (CCl_4_)-induced LF mice (He et al., 2020). Consistent with this, downregulation of RXRα mRNA has also been reported during activation of HSCs in rats with advanced LF induced by bile duct ligation and CCl_4_. In contrast, overexpression of RXRα in HSCs *in vitro* can inhibit the secretion of α-smooth muscle actin (α-SMA) and collagen I in HSCs, while *in vivo* overexpression of RXRα could resolve LF in mice, suggesting that this nuclear receptor plays a key role in the activation of HSCs and in LF (Ohata et al., 1997; Wang et al., 2011). Indeed, our previous RNA-seq study reported that miR-654-5p expression is significantly upregulated, while RXRα expression is downregulated in culture-activated human primary HSCs *in vitro* (Supplementary Figure S1). Therefore, in the current study, we hypothesized that miR-654-5p participates in the activation and proliferation of HSCs through the negative regulation of RXRα, thereby promoting LF.

## Materials and Methods

### Isolation of Primary Human HSCs, Cell Culture and Stimulation

Liver tissues were obtained intraoperatively from patients undergoing orthotopic liver transplantation or surgical liver resection for primary biliary cirrhosis, primary sclerosing cholangitis, and HBV-related cirrhosis. Demographic and clinical characteristics, laboratory indices, and disease statuses of the patients are shown in Supplementary Table S1. The distance from the obtained liver tissue to the edge of the lesion was at least 5 cm. Written informed consent was obtained from the patients for use of their tissues for research purposes, according to the ethical guidelines of the First Hospital of Jilin University (NO. 2019-356). Subsequently, primary human HSCs were isolated from the wedge sections of human livers using the density gradient centrifugation method (Werner et al., 2015). In brief, collagenase IV (Sigma-Aldrich, St. Louis, MO, United States) was used to perfuse and digest the liver tissue, and a hepatic cell suspension was obtained after blunt separation. Primary hepatocytes (HCs) were separated after centrifugation at 50 *g* for 5 min, and the supernatant was further centrifuged at 500 g for 5 min at 4°C. Using 8.5% Optiprep gradient medium (Stemcell, Vancouver, Canada), we removed other non-parenchymal cells, including liver sinusoidal endothelial cells and Kupffer cells. Primary HSCs, and the human immortalized hepatic stellate cell line (LX-2) (kindly provided by Dr. Zhengkun Tu) were maintained in Dulbecco’s modified eagle’s medium (DMEM; Gibco, Waltham, MA, United States) supplemented with 10% fetal bovine serum (FBS; Gibco). For TGFβ1-induced activation, LX-2 cells were treated with TGF-β1 (5 ng/μL; R&D, United States) for 24 h after starvation. The cells were then incubated in a 5% CO_2_ incubator at 37°C.

### Immunofluorescence Staining

HSCs were fixed with 4% paraformaldehyde (Solarbio, Beijing, China) for 15 min at room temperature (25°C), washed with phosphate-buffered saline (PBS), and subsequently blocked with 2% bovine serum albumin for 30 min. HSCs were then incubated for 2 h at 37°C with a primary monoclonal anti-α-SMA antibody (1:200 dilution; Abcam, Cambridge, MA). After washing with PBS, the cells were incubated for 1 h at room temperature with a secondary polyclonal goat anti-rabbit IgG (H + L; 1:200; Earthox, San Francisco, United States). The negative control was obtained by not using the primary antibodies. After incubation with the above antibodies, cells were washed with PBS and counterstained with 4,6-diamidino-2-phenylindole (DAPI; Invitrogen, Carlsbad, CA, United States) for 3 min. Immunofluorescence staining was detected and photographed using a laser scanning microscope (Axiovert 100M; Zeiss, Jena, Germany) at 200× magnification.

### Histological Analyses

Mouse liver tissue sections were cut into 3 μm silces, and embedded in paraffin. Collagen deposition in liver tissue sections was localized using standard histological techniques with Masson’s trichrome staining. Each section was assessed under a light microscopic and photographed at 40× magnification.

### Animals

Male C57BL/6 mice (6 weeks old) were purchased from Charles River (Beijing, China). All mice were fed a standard rat chow diet and housed under a 12 h light/dark cycle. After acclimatization for 7 days, the mice were randomly divided into four groups: negative control (NC; n = 6), CCl_4_ group (n = 6), CCl_4_+AAV-NC group (n = 7), and CCl_4_+AAV-miR-654-5p group (n = 7). Adeno-associated virus serotype 8 (AAV8) particles encoding miR-654-5p (hereafter referred to as AAV- miR-654-5p) and control AAV particles (AAV-NC) were purchased from Hanbio, Shanghai, China, and were administered to the CCl4+AAV-miR-654-5p and CCl4+AAV-NC groups at a dose of 3 × 10^11^ viral genomes (vg) per animal *via* tail vein injection. After 1 week, the mice in the CCl_4_, CCl_4_+AAV-NC, and CCl_4_+AAV-miR-654-5p groups were intraperitoneally injected with a 10% CCl4 (Aladdin, China) dose at 1 ml/kg (diluted with edible olive oil before injection) three times per week. Mice in the NC group were similarly administered the same solvent. After 6 weeks, the mice were sacrificed, and their liver tissues were dissected. Blood samples were centrifuged, and serum was stored at −80°C. Additionally, a portion of the liver tissue samples were fixed in 4% formaldehyde, while the remaining sample was stored at −80°C until use. All experiments involving mice were conducted in accordance with the ethical guidelines of the Animal Ethics Committee of First Hospital of Jilin University (Approval NO. 20220002).

### Transient Transfection

Cells were transfected with 50 nM miR-654-5p mimics or mimics-NC (RiboBio, Guangzhou, China) using Lipofectamine 3,000 (Invitrogen) following the manufacturer’s protocol. Similarly, cells were transfected with pcDNA3.1-RXRα plasmid or its control plasmid pcDNA3.1-NC (Sangon Biotech).

### Western Blot Assay

Cells were lysed in RIPA buffer (Beyotime, Shanghai, China) containing PMSF (Solarbio). This assay was performed using standard western blotting techniques with the following primary antibodies: anti-RXRα (Abcam), anti-collagen I (Proteintech, Chicago, United States), anti-MMP2 (Proteintech) and anti-tubulin (YTHX Biotechnology, Beijing, China).

### Quantitative Reverse‐transcription Polymerase Chain Reaction (qRT-PCR)

Total RNA was extracted from the cells/liver tissues using Eastep™ Total RNA Extraction Kit (Promega, Madison, United States) and miRNAs were extracted from the cells/liver tissues using the EasyPure miRNA Kit (TransGen, Beijing, China) following the manufacturer’s instructions. Complementary DNA (cDNA) was synthesized using TransScript One-Step gDNA Removal and cDNA Synthesis SuperMix (TransGen) to detect mRNA. cDNA was generated using a Ribo SCRIPT™ Reverse Transcription kit (RiboBio) to detect miRNA. Quantitative real-time polymerase chain reaction (qRT-PCR) was performed using PerfectStart™ Green qPCR SuperMix (TransGen). β-actin was used as an mRNA control. U6 was used as a reference miRNA control. The primers for U6/miR-654-5p were obtained from RiboBio Co., Ltd. All other qPCR primers used are listed in Table 1 qPCR was performed using the Agilent Mx3005P Real-Time PCR System (Applied Biosystems, Foster City, CA).

**TABLE 1:** Primers used for the real‐time polymerase chain reaction.

Genes	Forward (5′-3′)	Reverse (5′-3′)
has-β-actin	CAC​CAT​TGG​CAA​TGA​GCG​GTT​C	AGG​TCT​TTG​CGG​ATG​TCC​ACG​T
has-col1α1	GAG​GGC​CAA​GAC​GAA​GAC​ATC	CAG​ATC​ACG​TCA​TCG​CAC​AAC
has-MMP2	AGC​GAG​TGG​ATG​CCG​CCT​TTA​A	CAT​TCC​AGG​CAT​CTG​CGA​TGA​G
has-α-SMA	CTA​TGC​CTC​TGG​ACG​CAC​AAC​T	CAG​ATC​CAG​ACG​CAT​GAT​GGC​A
has-RXRα	TTG​CCA​AGC​AGC​CGA​CAA​ACA​G	AAG​GAG​GCG​ATG​AGC​AGC​TCA​T
mmu-col1α1	CCT​CAG​GGT​ATT​GCT​GGA​CAA​C	CAG​AAG​GAC​CTT​GTT​TGC​CAG​G
mmu-α-SMA	TGC​TGA​CAG​AGG​CAC​CAC​TGA​A	CAG​TTG​TAC​GTC​CAG​AGG​CAT​AG
mmu-MMP2	CAA​GGA​TGG​ACT​CCT​GGC​ACA​T	TAC​TCG​CCA​TCA​GCG​TTC​CCA​T
mmu-RXRα	GTG​AAA​GAT​GGG​ATT​CTC​CTG​GC	GTC​ACG​CAT​CTT​AGA​CAC​CAG​C

### Luciferase Reporter Assay

The wild-type (WT) or mutant (MUT) RXRα 3ʹ UTR was synthesized and subcloned into the pmirGLO Dual-Luciferase miRNA Target Expression Vector (Promega). HEK293 cells were co-transfected with miR-654-5p mimics and pGLO‐WT‐RXRα or pGLO‐MUT‐RXRα using Lipofectamine 3,000. Luciferase activity was measured 48 h after the co-transfection using the Dual-Luciferase Reporter Assay System (Promega) following the manufacturer’s instructions. Relative luciferase activity was calculated by normalizing firefly luciferase activity to Renilla luciferase activity.

### Cell Counting Kit-8 (CCK-8) Assay

LX-2 cells were seeded into 96-well plates (3,000 cells/well) and cultured in serum-free DMEM after transfection; the medium was replaced every 48 h. CCK-8 solution (10 uL; Beyotime, Shanghai, China) was added to each well at 24, 48, 72, 96, 120, 144, and 168 h after transfection, respectively. Absorbance was measured at 450 nm using a microplate reader (Thermo Fisher Scientific, Waltham, MA, United States).

### Flow Cytometry Analysis

To detect cell apoptosis, LX-2 cells were cultured in serum-free DMEM for 5 days after transfection to induce apoptosis by starvation. Trypsin was used to digest the cells for flow cytometry analysis. Cell apoptosis was measured by staining cells with PE Annexin V and 7-AAD using a PE Annexin V Apoptosis Detection Kit (BD Biosciences, Franklin Lakes, NJ, United States) for 15 min at room temperature in the dark. Apoptosis was detected using a FACS Canto flow cytometer (BD).

To detect the purity of isolated HSCs, freshly isolated HSCs were washed and counted They were then incubated with CD68 and CD146 antibodies (BD Biosciences, San Jose, CA) in the dark at room temperature for 30 min. In addition, other HSCs were cultured *in vitro* for 14 days and then collected. After washing, cells were fixed and permeabilized with the BD Cytofix/Cytoperm™ Fixation/Permeabilization kit (BD) according to the manufacturer’s introductions. Fixed cells were further incubated with an α-SMA antibody (R&D). After washing, labeled cells were resuspended and analyzed by flow cytometry. Flowjo was used to analyze the flow data.

### ALT, AST and Hydroxyproline Measurements

The levels of serum alanine aminotransferase (ALT), aspartate aminotransferase (AST) and hydroxyproline (Hyp) were measured in the mice using ALT, AST and Hyp measuring reagent kits (Nanjingjiancheng, Nanjing, China) according to the manufacturer’s instructions.

### Statistical Analysis

Data are presented as mean ± standard deviation (SD) of at least three independent experiments. The paired *t*-test was used to analyze the differences in mRNA or miRNA expression from qRT-PCR results, and *p < 0.05, **p < 0.01, ***p < 0.001, and ****p < 0.0001 from Prism 8.0.1 software (GraphPad Software, San Diego, CA) were defined as statistically significant.

## Results

### Identification of Isolated HSCs

**FIGURE 1:**
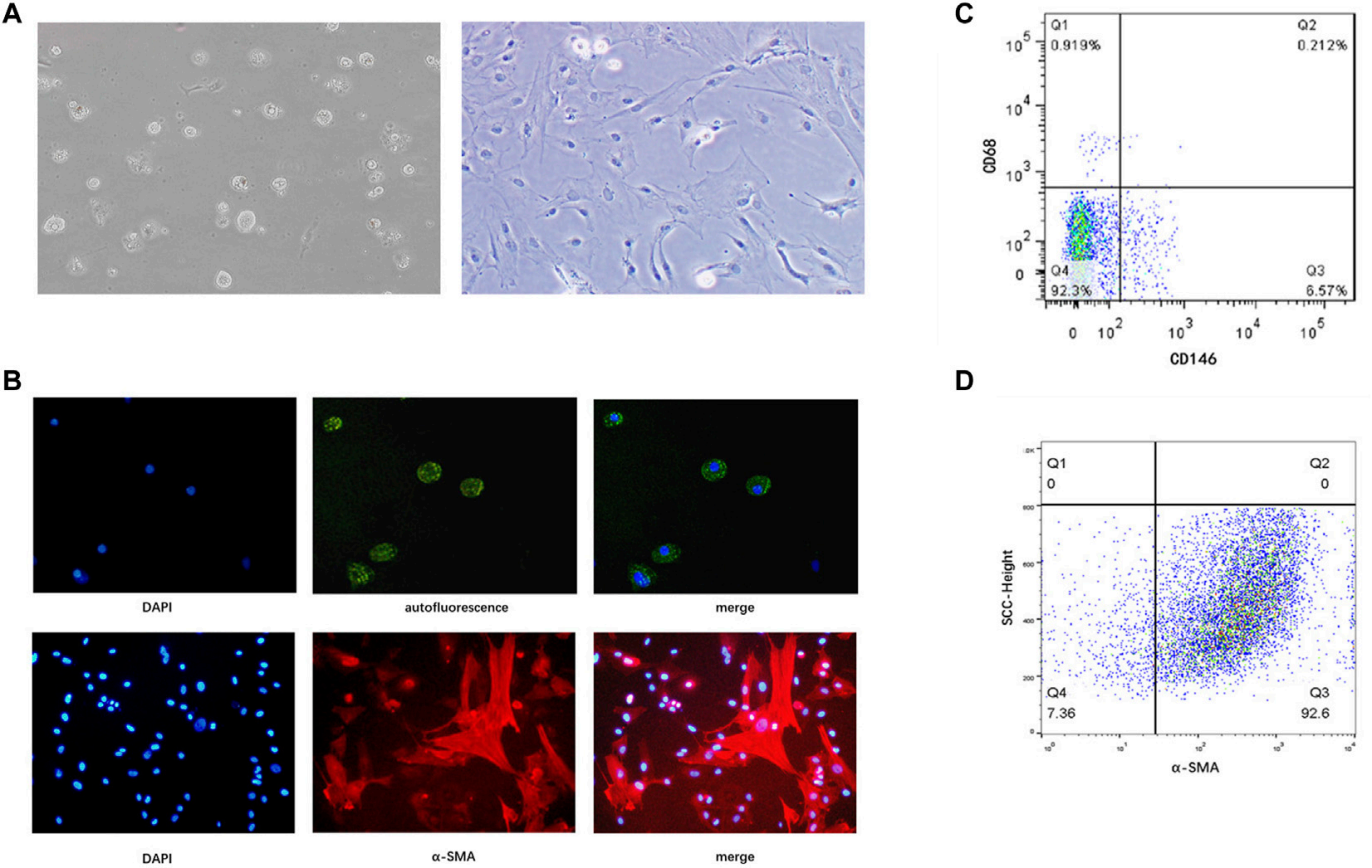
Identification of primary human HSCs isolated from liver tissue specimens. **(A)** Freshly isolated primary HSCs (1 d) and HSCs cultured *in vitro* for 14 days in a light microscopic field at 200× magnification. **(B)** Spontaneous fluorescence (green) and DAPI (blue) staining under a laser scanning microscope in a light microscopic field (upper panels, original magnification: ×200), and immunofluorescence staining with an anti-α-SMA antibody (red) and DAPI (blue; lower panels, original magnification: ×200). **(C)** Purity of freshly isolated human HSCs determined by flow cytometry. CD146 identifies liver endothelial cells, and CD68 identifies macrophages. Cells that express neither CD68 nor CD146 are considered HSCs **(D)** Purity of culture-induced activated HSCs determined by flow cytometry. HSCs, hepatic stellate cells.

Although there are no known specific markers for quiescent human HSCs, these cells store several cytoplasmic retinoid droplets rich in vitamin A. Therefore, we detected spontaneous fluorescence of these lipid droplets in freshly separated (1 d) HSCs. HSCs were cultured in plastic dishes for 14 days to induce spontaneous cellular activation. The activated HSCs underwent morphological changes and expressed α-SMA (Figures 1A,B). Moreover, since HSCs are similar in size to liver endothelial cells and macrophages, it is likely that the layer obtained following density gradient centrifugation contained all three of these cell subsets. Therefore, we identified liver endothelial cells *via* CD146 and macrophages *via* CD68. The remaining CD68^–^ CD146^–^ cells were considered to be HSCs. The results showed that the purity of freshly isolated HSCs was >90% (Figure 1C). Subsequently, we labeled HSCs with α-SMA and analyzed them *via* flow cytometry, confirming that the purity of isolated HSCs was >90% (Figure 1D).

### MiR-654-5p is Significantly Upregulated While RXRα is Downregulated During Natural Activation of HSCs and in TGF-β1-Treated LX-2 Cells

**FIGURE 2:**
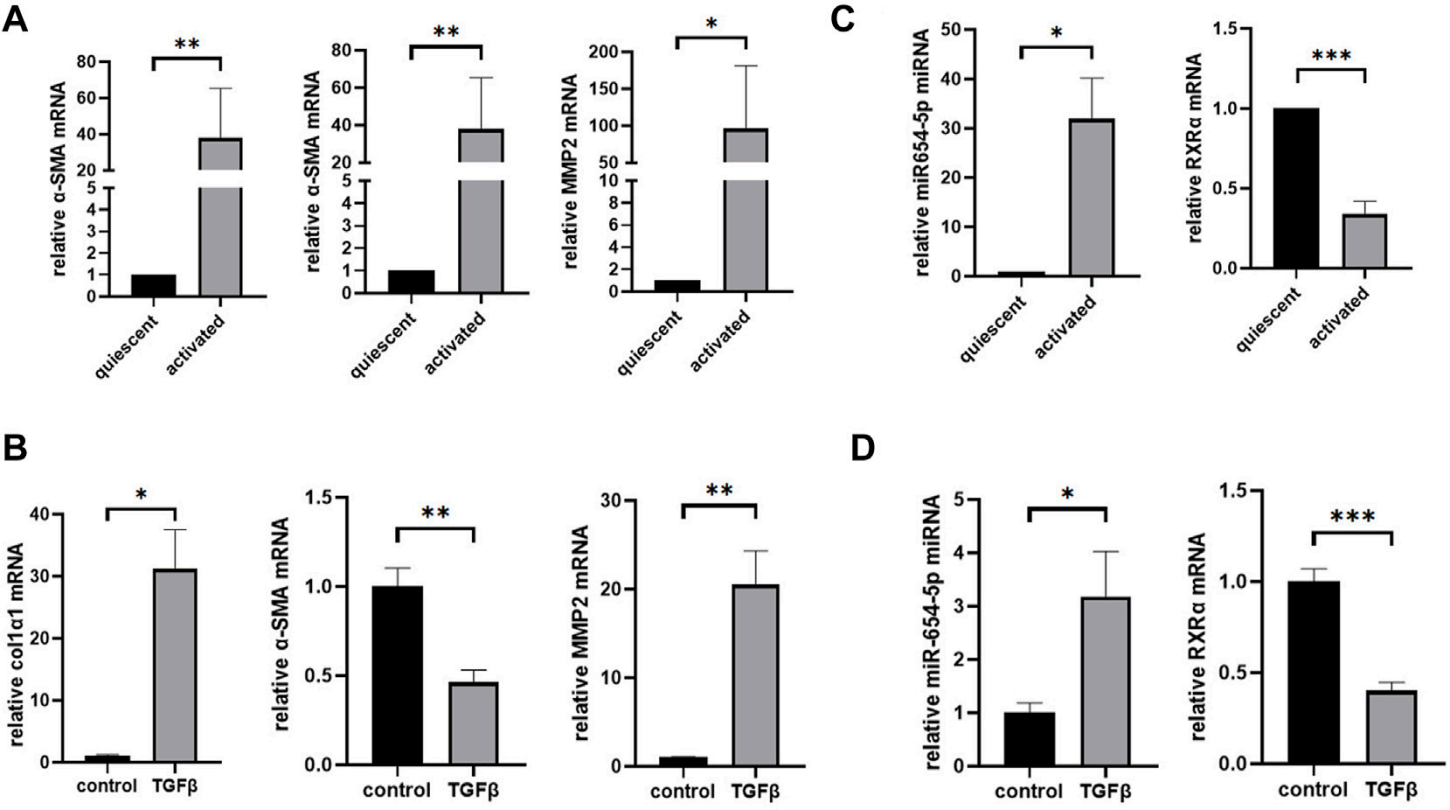
MiR-654-5p expression levels increase while RXRα mRNA expression levels decrease in culture-activated HSCs and TGFβ1-treated LX-2 cells. **(A)** col1α1, α-SMA, and MMP2 mRNA expression levels in culture-activated HSCs versus quiescent HSCs. Quantitative polymerase chain reaction analyses were performed to quantify mRNA expression levels with β-actin as a loading control **(B)** col1α1, α-SMA, and MMP2 mRNA expression levels in TGFβ1-treated LX-2 cells versus the control group. **(C)** MiRNA-654-5p and RXRα expression levels in culture-activated HSCs versus quiescent HSCs. Quantitative polymerase chain reaction analyses were performed to quantify miRNA expression levels with U6 as a loading control **(D)** MiRNA-654-5p and RXRα expression levels in TGFβ1-treated LX-2 cells versus in the control group. Error bars represent mean ± SEM of at least three experiments. *p < 0.05, **p < 0.01, ***p < 0.001 and ****p < 0.0001. HSCs, hepatic stellate cells.

To confirm the spontaneous activation of HSCs following *in vitro* culture, the expression levels of activation-related genes in HSCs were measured. After 14 days of *in vitro* culture, the mRNA expression levels of collagen type 1-α1 (col1α1), α-SMA, and matrix metalloproteinase 2 (MMP2) were significantly upregulated compared to those at on day 1 (Figure 2A). Similarly, after 48 h of TGFβ1 stimulation, the mRNA expression levels of col1α1 and MMP2 in LX-2 cells were significantly upregulated, while that of α-SMA decreased (Figure 2B). After *in vitro* culturing for 14 days, miR-654-5p and RXRα expression was evaluated in activated primary HSCs. Our results showed that the relative expression of miR-654-5p was significantly increased in activated HSCs compared to quiescent HSCs (freshly isolated; p < 0.05), while the relative expression of RXRα decreased (*p* < 0.001; Figure 2C). In addition, we treated LX-2 cells with TGF-β1, an HSCs activator. A similar trend was observed in TGF-β-induced LX-2 cells compared to the NC group (*p* < 0.05 and *p* < 0.001, respectively; Figure 2D).

### Upregulation of miR-654-5p Promotes Activation and Proliferation of LX-2 Cells While Inhibiting Their Apoptosis

**FIGURE 3:**
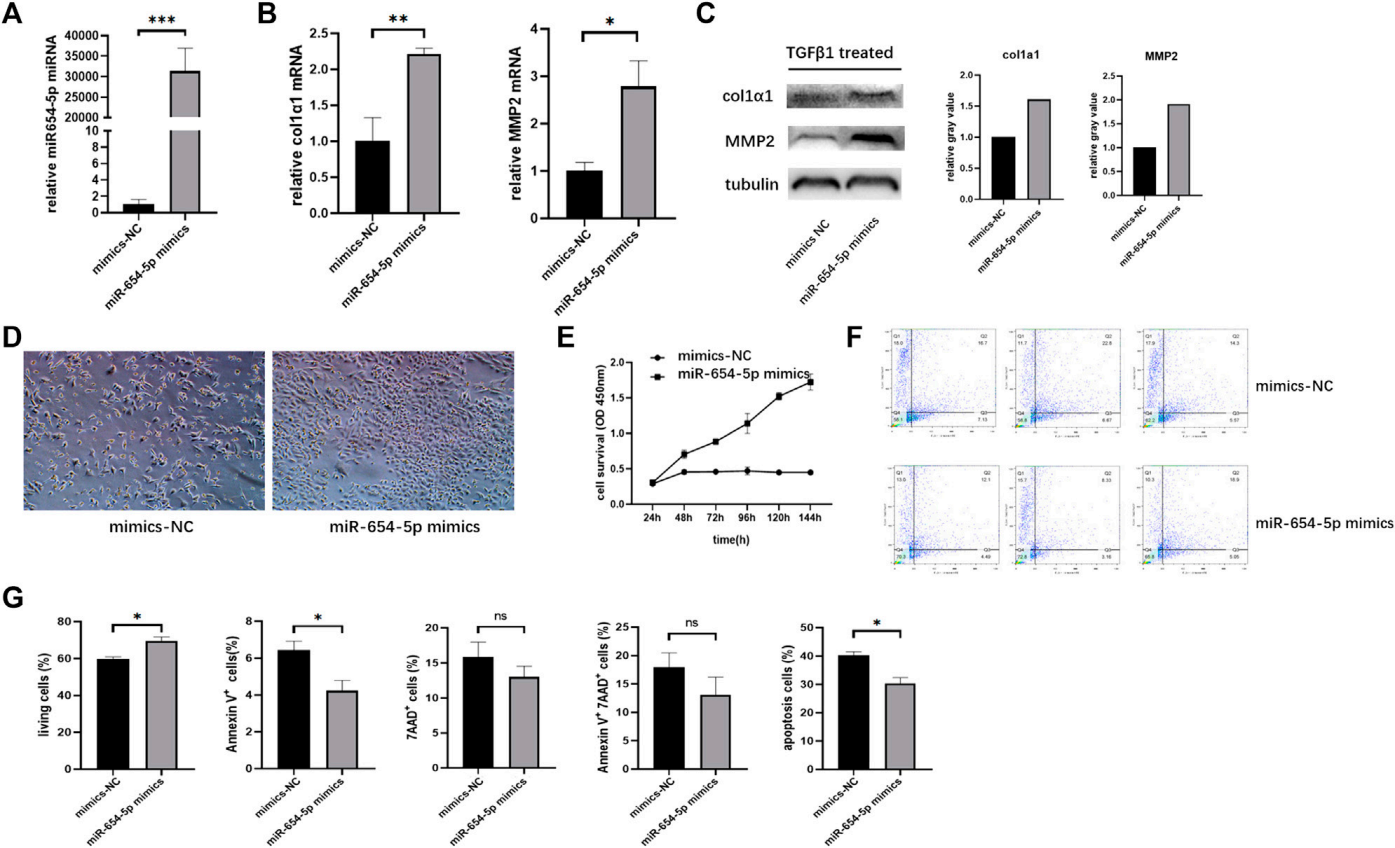
Upregulation of miR-654-5p expression promotes activation and proliferation of LX-2 cells while inhibiting their apoptosis. **(A)** MiRNA-654-5p expression levels in LX-2 cells transfected with miR-654-5p mimics versus LX-2 cells transfected with mimics-NC **(B)** col1α1 and MMP2 mRNA expression levels in miR-654-5p overexpressing LX-2 cells versus the control group. **(C)** Expression of col1α1 and MMP2 protein in miR-654-5p overexpressing LX-2 cells versus in control group **(D)** Quantity and morphology of LX-2 cells transfected with mimics-NC and LX-2 cells transfected with the miR-654-5p mimic (cells were photographed under light microscopic field, 40× magnification). **(E)** Effects of miR‐654‐5p mimics on the growth of LX-2 cells. Cells were transfected with the hsa‐miR‐654‐5p mimics. CCK-8 assay evaluated cell survival **(F)** Effects of the hsa‐miR‐654‐5p mimics on apoptosis of LX-2 cells **(G)** Flow cytometric analysis of apoptotic cells ratio. Cells were transfected with the hsa‐miR‐654‐5p mimics and cultured in an FBS-free medium for 5 days. Error bars represent mean ± SEM of at least three independent experiments. *p < 0.05, **p < 0.01, and ***p < 0.001; NS, *p* > 0.05.

To explore the function of miR‐654‐5p in LF, we modulated the expression of miR‐654‐5p by transfecting LX-2 cells with mimics. First, we overexpressed miR‐654‐5p in LX-2 cells by transfection with miR-654-5p mimics (Figure 3A) and found that overexpression of miR‐654‐5p promoted the mRNA expression levels of HSC activation markers col1α1 and MMP2 compared to the control group (mimics-NC; Figure 3B; *p* < 0.01 and *p* < 0.05). Subsequently, LX-2 cells were treated with TGFβ1 after miR-654-5p mimics/mimics-NC transfection. The resulting expression levels of col1α1 and MMP2 proteins were increased in miR-654-5p-overexpressing LX-2 cells (Figure 3C). These results validated the functional relevance of miR‐654‐5p in the activation of HSCs *in vitro*.

LX-2 cells transfected with the miR-654-5p mimic were then starved in an FBS-free medium for 5 days. The resulting quantity and morphology of LX-2 cells differed from those of the NC group (Figure 3D). Moreover, the CCK‐8 assay results showed that overexpression of miR‐654‐5p significantly promoted the proliferation of LX-2 cells (Figure 3E).

In addition, flow cytometry analysis results suggested that LX-2 cells treated with the miR‐654‐5p mimic exhibited changes in apoptosis compared to mimic-NC. Specifically, three repeated experiments showed that Annexin V-positive cells were significantly increased after miR-654-5p transfection; therefore, miR-654-5p reduced early apoptosis of LX-2 cells (Figures 3F,G). Overall, these results indicate that upregulation of miR‐654‐5p promotes the activation and proliferation of HSCs while inhibiting apoptosis.

### MiR-654-5p Directly Targets RXRα

**FIGURE 4:**
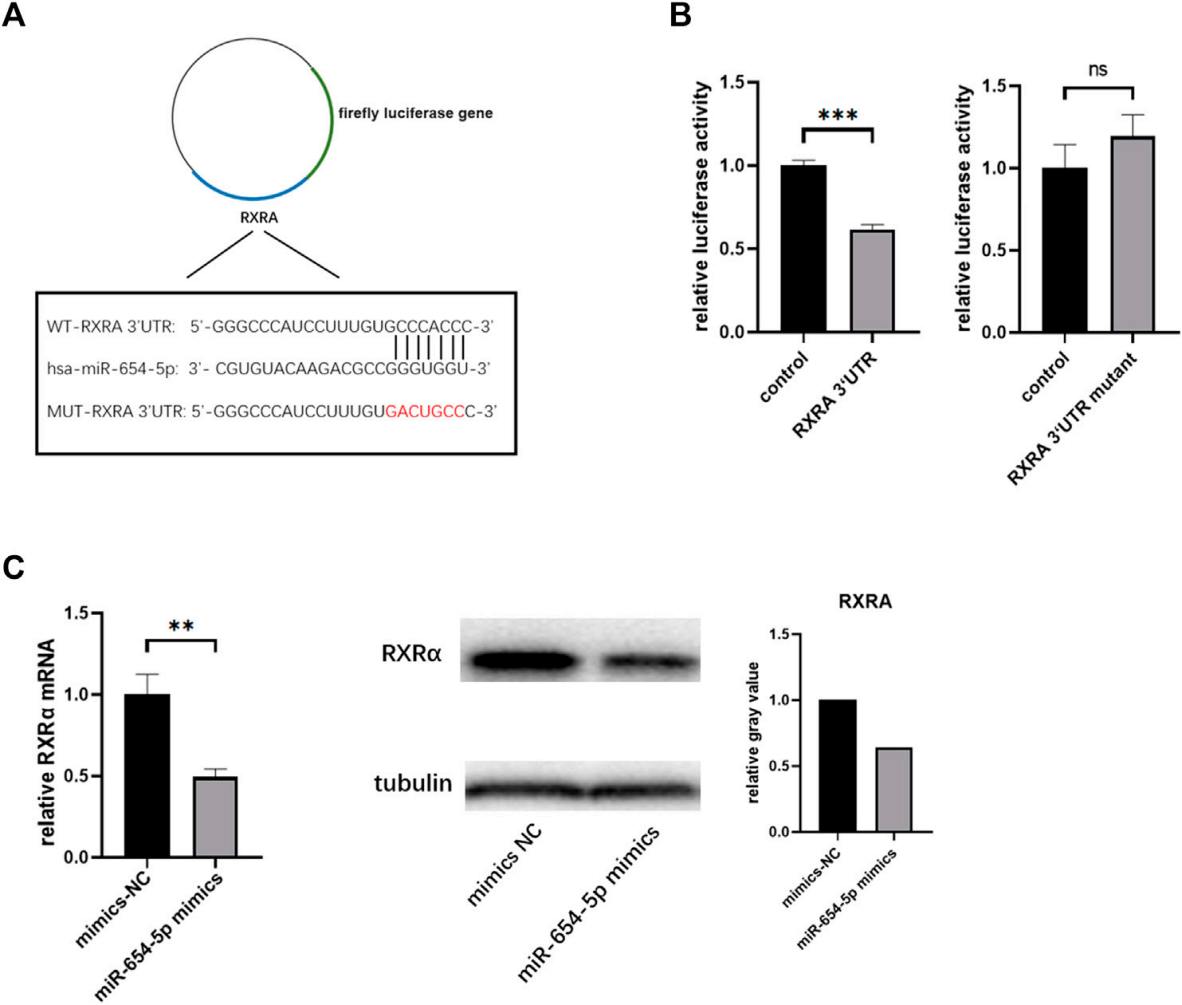
RXRα serves as a direct target of hsa‐miR‐654‐5p. **(A)** Binding region between hsa‐miR‐654‐5p and RXRα, and the luciferase reporter constructs containing the WT-RXRα or MUT-RXRα sequence **(B)** WT-RXRα or MUT-RXRα were co-transfected into HEK-293T cells with the hsa‐miR‐654‐5p mimic or the corresponding negative control **(C)** RXRα mRNA and protein expression levels in LX-2 cells. Grey values are measured through ImageJ. Cells were transfected with the hsa‐miR‐654‐5p mimic or their corresponding negative controls. Error bars represent mean ± SEM of at least three independent experiments. **p < 0.01, ***p < 0.001; ns, *p* > 0.05. MUT, mutant; WT, wild type.

To identify the potential mRNAs targeted by miR-654-5p, bioinformatics analysis using TargetScan (http://www.targetscan.org/vert_71/) and miRwalk (http://mirwalk.umm.uni-heidelberg.de/) databases were carried out. Based on our lab RNA-seq data from the previous period, RXRα was noted to be a candidate involved in miR‐654‐5p regulation of HSCs. The speculative binding region between hsa‐miR‐654‐5p and RXRα, based on TargetScan results, is shown in Supplementary Figure S2. A dual-luciferase reporter assay was then used to validate the association between the two. To this end, luciferase reporter plasmids of WT‐RXRα- and MUT‐RXRα 3ʹ UTR were constructed and are shown in Figure 4A. Co-transfection of the luciferase reporter plasmid containing WT‐RXRα with miR‐654‐5p mimics in HEK‐293T cells decreased reporter activity. Conversely, the luciferase reporter plasmid co-transfection containing MUT‐RXRα with miR‐654‐5p mimics did not alter the luciferase activity (Figure 4B). In addition, the levels of both RXRα mRNA and protein were markedly inhibited by miR‐654‐5p upregulation in LX-2 cells (Figure 4C). These findings indicate that miR-654-5p directly targets RXRα.

### RXRα Overexpression Rescues the Effect of miR‐654‐5p on LX-2 Cells

**FIGURE 5:**
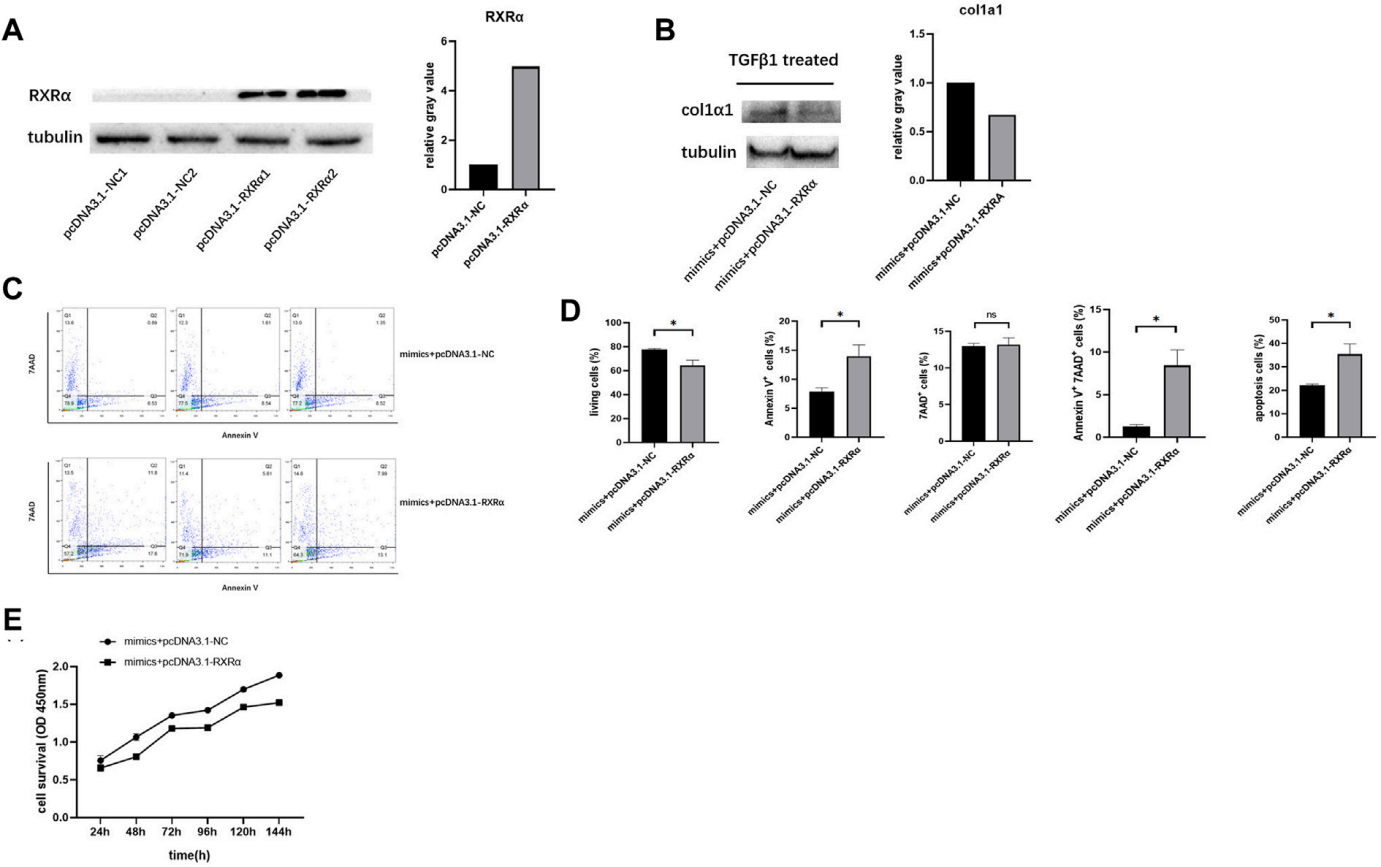
RXRα overexpression rescues the effect of miR‐654‐5p on LX-2 cells. **(A)** Expression level of RXRα protein in cells transfected with pcDNA3.1-RXRα plasmid or the corresponding negative control, grey values are measured through ImageJ **(B)** Expression level of col1α1 protein in LX-2 cells co-transfected with miR-654-5p mimics and pcDNA3.1-NC/pcDNA3.1-RXRα plasmid, grey values are measured through ImageJ. **(C)** Combined effects of the hsa‐miR‐654‐5p mimic and pcDNA3.1-NC/pcDNA3.1-RXRα on LX-2 cell apoptosis **(D)** Flow cytometric analysis to determine the ratio of apoptotic cells. After co-transfection with the hsa‐miR‐654‐5p mimic and pcDNA3.1-NC/pcDNA3.1-RXRα, cells were cultured in an FBS-free medium for 5 days. **(E)** Cell proliferation evaluated via CCK-8 assay after co-transfection with the miR‐654‐5p mimic and pcDNA3.1-NC/pcDNA3.1- RXRα. Error bars represent mean ± SEM of at least three independent experiments. *p < 0.05.

To verify that miR-654-5p regulates the activation, proliferation and apoptosis of HSCs by targeting RXRα, we transfected LX-2 cells with pcDNA3.1-RXRα plasmid to ectopically overexpress RXRα and confirmed the elevated levels of RXRα (Figure 5A), LX-2 cells were then co-transfected with miR-654-5p mimics and pcDNA3.1-RXRα/pcDNA3.1-NC. Subsequently, the transfected cells were treated with TGFβ1 to induce col1α1 expression. The results of western blotting showed that the overexpression of RXRα suppress the miR-654-5p mimics-induced expression of col1α1 protein (Figure 5B).

The co-transfected cells were also obtained to assess apoptosis using flow cytometry analysis. Results showed that following co-transfection with pcDNA3.1-RXRα and miR‐654‐5p mimics, the proportion of LX-2 cells undergoing early apoptosis had increased compared to those transfected with pcDNA3.1-NC and miR‐654‐5p mimics (Figures 5C,D). Similarly, CCK-8 assay results showed a decline in the proliferation in cells co-transfected with pcDNA3.1-RXRα and miR‐654‐5p mimics (Figure 5E).

### MiR-654-5p Is Increased in CCl_4_-Induced LF, and miR-654-5p Overexpression Aggravates LF in Mice

**FIGURE 6:**
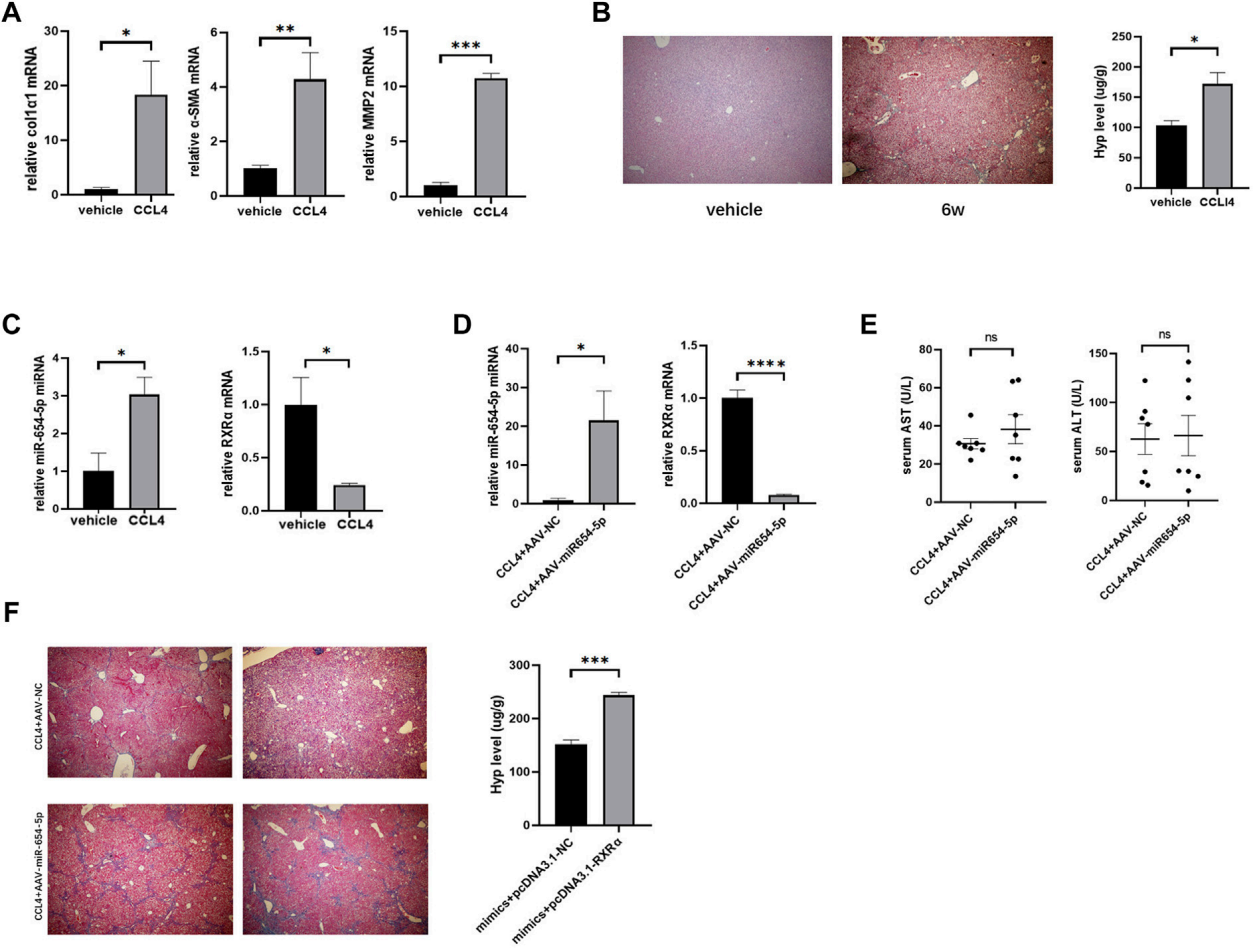
MiR-654-5p expression level is increased causing an aggravating effect in CCl4-induced mouse LF, owing to RXRα targeting. **(A)** col1α1, α-SMA, and MMP2 mRNA expression levels in the livers of CCl_4_-treated mice versus vehicle. Quantitative polymerase chain reaction analyses were performed to quantify mRNA expression levels, with β-actin as a loading control **(B)** After treating with olive oil control (vehicle) or CCl4 for 6 weeks, Masson’s trichrome images (40× magnification) and hydroxyproline measurement were used to evaluate liver fibrosis. **(C)** MiR-654-5p and RXRα levels in liver tissues. U6 was used as a loading control for miR-654-5p **(D)** Expression levels of miR-654-5p and RXRα, as quantified *via* qPCR analysis of liver tissue samples. **(E)** Serum ALT and AST levels quantified to determine the extent of liver damage in the different groups **(F)** Liver fibrosis evaluated *via* Masson’s trichrome staining and hydroxyproline measurement. Error bars represent mean ± SEM of at least three in dependent experiments. *p < 0.05, **p < 0.01, ***p < 0.001 and ****p < 0.0001; ns, *p* ≥ 0.05. CCl_4_, carbon tetrachloride; ALT, alanine aminotransferase; AST, aspartate aminotransferase.

To further investigate the role of miR-654-5p *in vivo*, we established a CCl_4_-induced LF mouse model. The mRNA levels of α-SMA, col1α1, and MMP2, were higher in the livers of CCl_4_-treated mice than in those of control mice (Figure 6A). Masson’s trichrome staining further revealed increased ECM deposition in the livers of CCl4-treated mice compared to those of control mice. In addition, Hyp content was upregulated in the liver tissues of mice treated with CCl_4_ (Figure 6B). These results confirmed that CCl_4_ induced LF in the mouse model.

Subsequently, the levels of mmu-miR-654-5p and mmu-RXRα between the CCl_4_ model and NC groups were estimated. Results showed that mmu-miR-654-5p expression was upregulated in fibrotic mouse, while mmu-RXRα mRNA level was consistently downregulated (Figure 6C). The targeting relationship between mmu-miR-654-5p and mmu-RXRα was then predicted using TargetScan (Supplementary Figure S3). To assess the delivery of AAV into the liver, the level of miR-654-5p was quantified in mouse livers by qRT-PCR analysis. Expression of miR-654-5p was significantly upregulated in the CCl_4_+AAV-miR-654-5p group compared to that in the CCl_4_+AAV-NC group. Consistent with this, the mRNA expression of RXRα was decreased (Figure 6D). In addition, serum ALT and AST levels were compared between the CCl_4_+AAV-miR-654-5p and CCL4+AAV-NC groups. No significant differences were observed in AST and ALT content levels between the two groups (Figure 6E). Finally, we also observed a significant increase in fibrosis within AAV-miR-654-5p overexpressing livers, as evidenced by increased collagen deposition in the liver, detected *via* Masson’s trichrome staining, and increased Hyp levels (Figure 6F).

## Discussion

In the current study, our findings showed that miR-654-5p expression was upregulated in culture-induced activated human primary HSCs and TGF-β1-stimulated LX-2 cells, suggesting that miR-654-5p is, at least partially, involved in LF development. Furthermore, transfection of cells with the miR-654-5p mimic significantly induced ECM synthesis, upregulated col1α1 expression, and promoted HSC proliferation, while inhibiting the apoptosis of HSCs. In addition, we found that miR-654-5p regulates profibrotic genes expression and cellular biological functions through RXRα.

LF is characterized by excessive deposition of ECM components, in particular type I collagen (Bataller and Brenner, 2005; Friedman, 2008). HSCs convert from a resting to active phenotype and migrate to the damaged area where they produce ECM after liver injury (Tsuchida and Friedman, 2017). Meanwhile, MMPs regulate liver matrix degradation, with MMP2 defined as one of the most relevant MMPs for degrading the normal liver matrix. Indeed, MMP2 is significantly upregulated in activated HSCs during liver fibrosis progression, regulating the degradation of the normal liver matrix and further promoting LF (Arthur, 2000). Col1α1 and MMP2 are primarily produced by HSCs during LF (Barcena-Varela et al., 2019). In this study, the overexpression of miR-654-5p promoted the expression of these fibrogenic genes in LX-2 cells, suggesting that miR-654-5p promotes the progression of LF.

Numerous studies have shown that miRNAs play key roles in HSC activation and LF. For example, miR-188-5p induces the activation and proliferation of HSCs, subsequently aggravating LF (Riaz et al., 2021). Meanwhile, miR-15b and miR-16 limit HSC proliferation and the fibrogenic response (Ma et al., 2021), whereas miR-34c promotes HSC activation and LF (Li et al., 2021c). Additionally, miR-494-3p attenuates HSC activation and induces apoptosis in LF (Li et al., 2021d). Moreover, miR-29a-3p suppresses HSC proliferation by targeting PIK3R3 (Fu et al., 2020).

Several studies have also investigated the effect of RXRα on HSC activation and the regulation of HSC function (Ohata et al., 1997; Vogel et al., 2000; He et al., 2020). RXRα inhibits HSC proliferation (Sharvit et al., 2013) and regulates apoptosis in certain cell types, including pancreatic beta cells (Chen et al., 2019) and mouse hippocampal cells (Litwa et al., 2016). Therefore, further investigation into RXRα-related regulation of HSCs may provide new perspectives for LF treatment.

Studies have shown that certain miRNAs participate in the LF process by targeting RXRα. For example, miR-34a expression is upregulated in the fibrotic liver, and miR-34a regulates the expression of downstream genes by targeting RXRα (Oda et al., 2014). Moreover, Ji et al. (Ji et al., 2009) found that miR-27a and 27b were upregulated in activated rat HSCs *in vitro*. Meanwhile, transfecting HSCs with anti-miR-27a and anti-miR-27b restored lipid droplets and inhibited the proliferation of HSCs by negatively regulating RXRα. To better understand the biological function of miR-654-5p in LF, we overexpressed miR-654-5p in LX-2 cells, and our *in vitro* results demonstrated that miR-654-5p directly targets RXRα. Moreover, overexpression of miR-654-5p induces HSCs to synthesize collagen by inhibiting RXRα during LF. We also observed a significant increase in miR-654-5p expression and downregulation of RXRα in the CCl_4_-induced fibrosis mouse model. Therefore, collectively these findings suggest that overexpression of miR-654-5p promotes LF progression in mice.

Several limitations were noted in this study. First, in addition to an miR-654-5p mimic, the miR-654-5p inhibitor was also used to treat LX-2 cells in this study. However, owing to the low transcription baseline of miR-654-5p in LX-2 cells, no significant effect was observed on its expression following the use of its inhibitor (this data is not shown in the paper). As an alternative, we overexpressed miR-654-5p in a murine model rather than inhibiting it. Second, α-SMA is a marker of myofibroblasts and is upregulated during HSC activation (myofibroblast-like cells) (Liu et al., 2019). However, in our study, the miR-654-5p mimic did not induce a significant change in the expression of α-SMA in LX-2 cells (data not shown). We speculate that the lack of detectable mRNA responses might be due to characteristics of LX-2, which is an immortalized HSC cell line and can be considered an activated phenotype of primary HSCs (Xu et al., 2005). In our study, although we controlled for the number of passages of LX-2 (<10), α-SMA mRNA in LX-2 cells had a very high baseline level of α-SMA mRNA, which made it difficult to increase during further activation of LX-2 cells. Furthermore, no significant changes were detected in the expression of α-SMA in LX-2 cells after treatment with TGF-β1.

In conclusion, the findings of the study show that miR-654-5p induces activation and proliferation of HSCs, while inhibiting their apoptosis. In addition, miR-654-5p aggravates LF by, at least in part, blocking RXRα. Therefore, our findings may provide a new treatment strategy for LF.t

## Data Availability

The original contributions presented in the study are included in the article/Supplementary Material, further inquiries can be directed to the corresponding authors.

## Supplementary Material

Supplementary Figure S1 MiR-654-5p and RXRα expression levels in culture-activated HSCs/quiescent HSCs according to previous RNA-sequencing data. Error bars represent mean ± SEM of experiments, **p < 0.01, and ***p < 0.001.

Supplementary Figure S2 The targeting relationship between miR-654-5p and RXRα in human predicted by the Targetscan database.

Supplementary Figure S3 The targeting relationship between miR-654-5p and RXRα in mouse predicted by the Targetscan database.
