# Supplementary figures and images for: miR-654-5p Contributes to the Activation and Proliferation of Hepatic Stellate Cells by Targeting RXRα
Source: Front Cell Dev Biol. 2022 Apr 6;10:841248. doi: 10.3389/fcell.2022.841248 (PMC9019757; doi:10.3389/fcell.2022.841248)

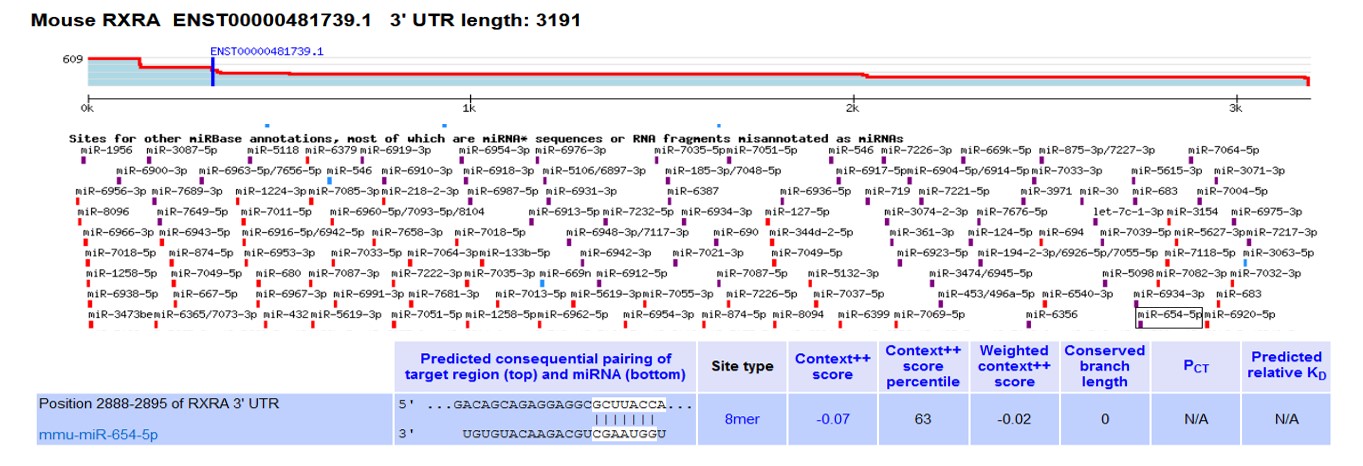

Supplement: Supplementary file 2 [file Image3.JPEG]

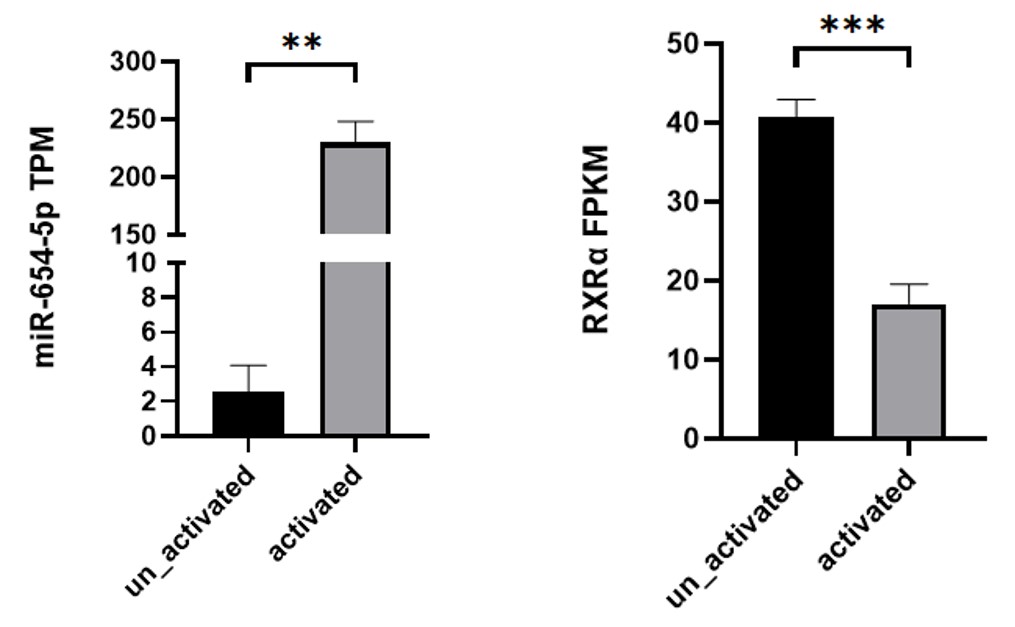

Supplement: Supplementary file 3 [file Image1.JPEG]

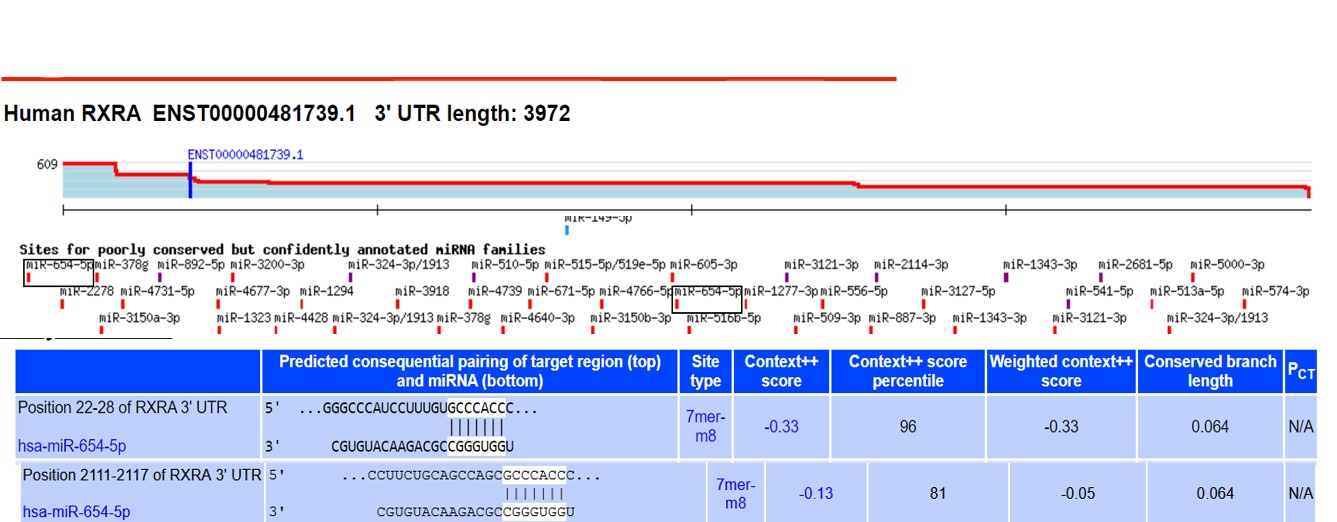

Supplement: Supplementary file 4 [file Image2.JPEG]
